# Supplementary material for: Impact of Synbiotic Intake on Liver Metabolism in Metabolically Healthy Participants and Its Potential Preventive Effect on Metabolic-Dysfunction-Associated Fatty Liver Disease (MAFLD): A Randomized, Placebo-Controlled, Double-Blinded Clinical Trial
Source: Nutrients. 2024 Apr 26;16(9):1300. doi: 10.3390/nu16091300 (PMC11085762; doi:10.3390/nu16091300)
Supplement: Supplementary file 1 [file nutrients-16-01300-s001.zip › nutrients-2934984-supplementary/Table S1.pdf]

Table S1: Baseline characteristics of energy and macronutrient, serotonin, and amino acid intake in the study population

	Total<br>n=86	SYN<br>n=45 (52.3%)	PLA<br>n=41 (47.7%)	*P* value
Energy (kcal/d)	2838.12 (1511.2, 5623.4)	2740.16 (1511.2,3918.1)	2943.25 (1538.5,5623.4)	0.205
CH (g/d)	304.80 (162.0,883.9)	289.04 (162.0,522.9)	321.72 (174.6,883.9)	0.135
Protein (g/d)	114.62 (52.1,284.7)	113.35 (52.1,284.7)	116.00 (60.9,223.0)	0.342
Fat (g/d)	119.80 (44.1,212.2)	115.42 (67.3,165.1)	124.51 (44.1,212.2)	0.372
Serotonin (μg/L)	116.55 (19.5,260.8)	118.1 (58.6,260.8)	114.46 (19.5,207.0)	0.882
Glutamic Acid (nmol/mL)	44.02 (8.8,108.4)	44.42 (8.8,108.8)	43.58 (15.9,85.9)	0.861
Asparagine (nmol/mL)	64.15 (44.3,100.8)	66.14 (44.3,100.8)	61.96 (46.3,94.1)	0.124
Serine (nmol/mL)	121.47 (77.5,176.6)	120.56 (83.2,176.6)	122.48 (77.5,168.6)	0.720
Glutamine (nmol/mL)	1013.97 (741.5,1357.1)	1005.22 (793.4,1341.9)	1023.57 (741.5,1357.1)	0.565
Histidine (nmol/mL)	65.62 (40.1,103.8)	65.81 (40.1,103.8)	65.41 (44.7,94.8)	0.586
Glycine (nmol/mL)	317.46 (193.1,541.0)	326.62 (197.3,541.0)	307.41 (193.1,486.2)	0.165
Threonine (nmol/mL)	132.67 (70.2,204.6)	137.87 (82.2,204.6)	126.97 (70.2,200.0))	0.100
Citrulline (nmol/mL)	34.1 (17.3,52.6)	35.45 (17.3,52.6)	34.11 (22.8,51.8)	0.378
Arginine (nmol/mL)	93.7 (52.0,159.6)	95.41 (52.0,155.4)	91.90 (60.1,159.6)	0.390
Alanine (nmol/mL)	351.86 (153.5,641.1)	353.81 (222.3,558.1)	349.71 (153.5,641.1)	0.825
Taurine (nmol/mL)	59.07 (37.0,98.0)	59.60 (37.0,92.0)	58.49 (39.9,98.0)	0.551
Tyrosine (nmol/mL)	70.46 (36.4,123.6)	68.45 (36.4,101.4)	72.67 (41.0,123.6)	0.156
Valine (nmol/mL)	324.57 (189.7,517.8)	325.29 (189.7,468.6)	323.77 (230.7,517.8)	0.583

Methionine (nmol/mL)	41.48 (27.6,66.9)	41.98 (29.5,61.8)	40.93 (27.6,66.9)	0.468
Tryptophan (nmol/mL)	65.69 (36.3,96.9)	66.60 (36.3,96.9)	64.70 (46.6,95.4)	0.394
Phenylalanine (nmol/mL)	71.13 (45.6,106.1)	70.76 (45.6,91.0)	71.53 (49.5,106.1)	0.764
Isoleucine (nmol/mL)	91.70 (61.2,152.7)	91.33 (61.2)	92.11 (64.0,152.7)	0.887
Ornithine (nmol/mL)	80.16 (37.1,140.8)	80.43 (37.1,140.8)	79.87 (45.9,128.9)	0.782
Leucine (nmol/mL)	173.02 (103.0,273.1)	172.11 (103.0,246.7)	174.03 (118.0,273.1)	0.681
Lysine (nmol/mL)	217.16 (129.8,395.6)	222.00 (129.8,304.3)	211.85 (137.7,395.6)	0.180
