# Supplementary material for: Impact of Synbiotic Intake on Liver Metabolism in Metabolically Healthy Participants and Its Potential Preventive Effect on Metabolic-Dysfunction-Associated Fatty Liver Disease (MAFLD): A Randomized, Placebo-Controlled, Double-Blinded Clinical Trial
Source: Nutrients. 2024 Apr 26;16(9):1300. doi: 10.3390/nu16091300 (PMC11085762; doi:10.3390/nu16091300)
Supplement: Supplementary file 1 [file nutrients-16-01300-s001.zip › nutrients-2934984-supplementary/Table S2.pdf]

Table S2 Characteristics of the one-year follow-up study population

	Total n=45	SYN n=23 (51.1%)	PLA n=22 (48.9%)	*P* value
Weight (kg)	81.10 ± 8.70	79.34 ± 9.25	82.94 ± 7.87	0.168
BMI (kg/m^2)	24.73 ± 2.37	24.82 ± 2.78	24.63 ± 1.90	0.791
Fat mass (%)	17.62 ± 3.99	17.75 ± 5.02	17.49 ± 2.61	0.829
Energy (kcal/d)	2544.19 ± 613.13	2394.66 ± 485.59	2658.55 ± 687.35	0.250
CH (g/d)	266.68 ± 79.84	236.01 ± 61.89	290.14 ± 85.62	0.065
Protein (g/d)	100.61 ± 26.35	96.98 ± 22.45	103.38 ± 29.35	0.660
Fat (g/d)	110.75 ± 33.68	110.72 ± 33.50	110.78 ± 34.84	0.917
TG (mg/dL)	96.64 ± 47.16	92.65 ± 48.82	100.82 ± 46.12	0.460
Total chol (mg/dL)	184.07 ± 38.19	180.43 ± 38.45	187.86 ± 38.44	0.520
LDL chol (mg/dL)	113.09 ± 29.71	110.35 ± 30.12	115.95 ± 29.70	0.533
HDL chol (mg/dL)	51.47 ± 11.04	51.74 ± 13.41	51.18 ± 8.17	0.867
AST (U/L)	25.60 ± 22.40	21.74 ± 5.11	29.64 ± 31.47	0.452
ALT (U/L)	22.51 ± 10.02	22.83 ± 9.50	22.18 ± 10.74	0.609
GGT (U/L)	20.27 ± 11.19	21.09 ± 12.77	19.41 ± 9.49	0.741
PAL (U/L)	69.38 ± 19.27	68.91 ± 22.60	69.86 ± 15.58	0.407
Glucose (mg/dL)	89.77 ± 7.17	89.61 ± 5.87	89.95 ± 8.51	0.876
Insulin (mU/L)	8.73 ± 4.40	8.64 ± 4.67	8.82 ± 4.21	0.742
HOMA-IR	1.99 ± 1.10	1.93 ± 1.08	2.05 ± 1.14	0.690
hs-CRP (mg/L)	4.40 ± 11.77	9.87 ± 19.78	1.37 ± 0.65	0.894
IL-6 (pg/mL)	2.70 ± 2.08	3.04 ± 2.85	2.41 ± 1.07	0.927
