# Supplementary material for: Impact of Synbiotic Intake on Liver Metabolism in Metabolically Healthy Participants and Its Potential Preventive Effect on Metabolic-Dysfunction-Associated Fatty Liver Disease (MAFLD): A Randomized, Placebo-Controlled, Double-Blinded Clinical Trial
Source: Nutrients. 2024 Apr 26;16(9):1300. doi: 10.3390/nu16091300 (PMC11085762; doi:10.3390/nu16091300)
Supplement: Supplementary file 1 [file nutrients-16-01300-s001.zip › nutrients-2934984-supplementary/Table S3.pdf]

Table S3: Change in gut microbiome composition in each stratified group.

	**Physiological Bodyfat Percentage (n=32)**			**Elevated Bodyfat Percentage (n=30)**		
Coefficients	*Estimates*	*CI*	*P Value*	*Estimates*	*CI*	*P Value*
(Intercept)	-0.13	-0.68 – 0.41	0.62	0.12	-0.42 – 0.66	0.655
Age	0.03	-0.37 – 0.43	0.88	0.28	-0.09 – 0.65	0.134
Fat Mass	-0.07	-0.33 – 0.48	0.71	-0.11	-0.47 – 0.24	0.520
Group [SYN]	0.26	-0.55 – 1.06	0.51	-0.10	-0.82 – 0.63	0.785
Baseline Microbial Diversity	-0.22	-0.75– 0.30	0.39	-1.27	-2.29 – -0.26	**0.016**
Baseline Microbial Diversity*Group [SYN]	0.15	-0.68 – 0.97	0.72	1.16	0.06 – 2.25	**0.039**
Marginal R2 / Conditional R2	0.046 / -0.137			0.328 / 0.188		
