# Supplementary figures and images for: Impact of Synbiotic Intake on Liver Metabolism in Metabolically Healthy Participants and Its Potential Preventive Effect on Metabolic-Dysfunction-Associated Fatty Liver Disease (MAFLD): A Randomized, Placebo-Controlled, Double-Blinded Clinical Trial
Source: Nutrients. 2024 Apr 26;16(9):1300. doi: 10.3390/nu16091300 (PMC11085762; doi:10.3390/nu16091300)

**Supplementary Figure S1:** Flow chart of the study population from randomization to analysis

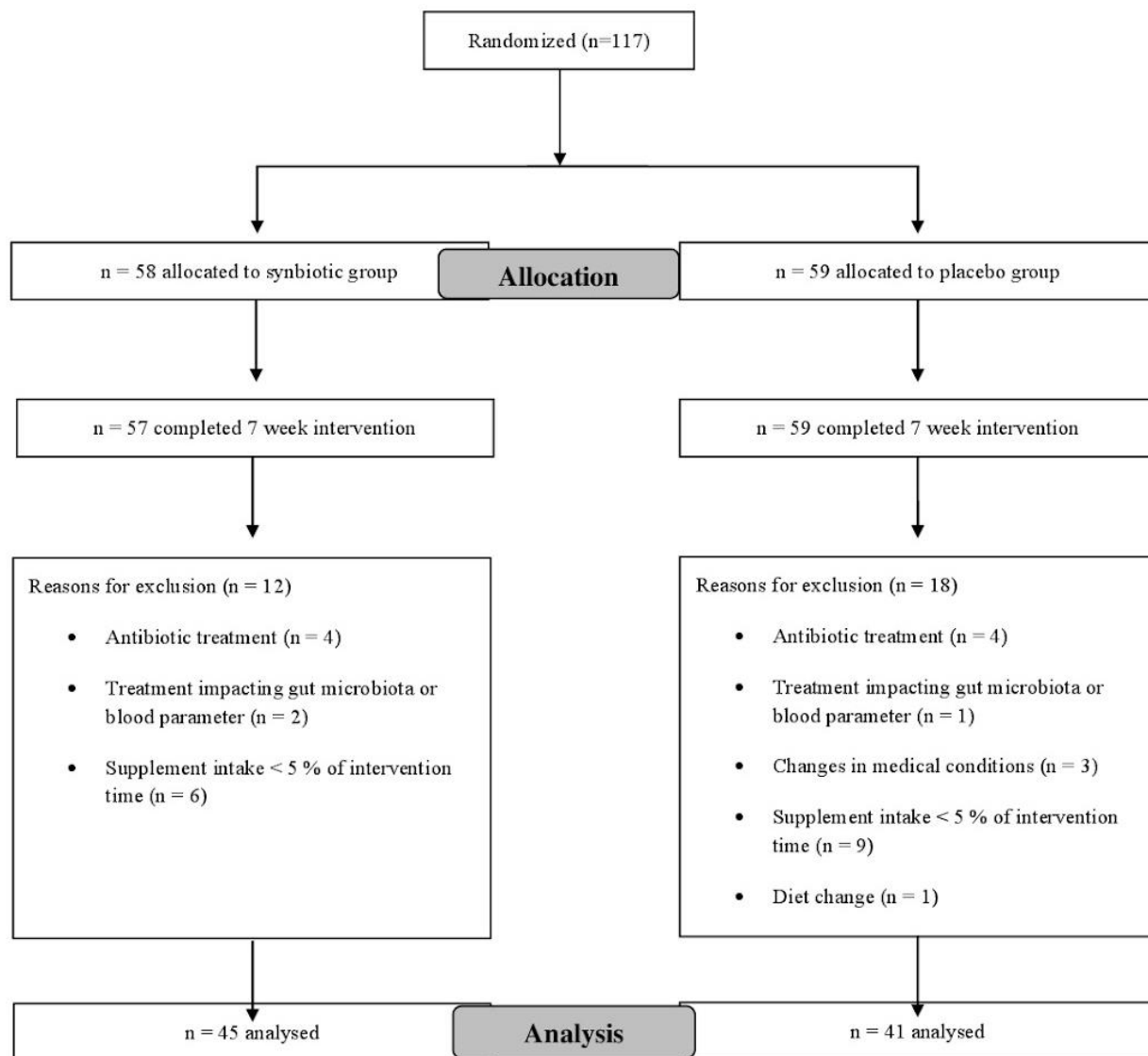

Supplement: Supplementary file 1 [file nutrients-16-01300-s001.zip › nutrients-2934984-supplementary/Figure S1.pdf]
